# Oxidation of Cysteinate Anions Immobilized in the Interlamellar Space of CaAl-Layered Double Hydroxide

**DOI:** 10.3390/ma14051202

**Published:** 2021-03-04

**Authors:** Zita Timár, Truong Ngoc Hung, Cora Pravda, Zoltán Kónya, Ákos Kukovecz, Pál Sipos, Gábor Varga, István Pálinkó

**Affiliations:** 1 Materials and Solution Structure Research Group and Interdisciplinary Excellence Centre, Institute of Chemistry, University of Szeged, Aradi Vértanúk tere 1, H-6720 Szeged, Hungary; zita.timar92@chem.u-szeged.hu (Z.T.); thuanhung1987@gmail.com (T.N.H.); palinko@chem.u-szeged.hu (I.P.); 2 Department of Organic Chemistry, University of Szeged, Dóm tér 8, H-6720 Szeged, Hungary; 3 Department of Applied and Environmental Chemistry, University of Szeged, H-6720 Szeged, Hungary; cora@chem.u-szeged.hu (C.P.); konya@chem.u-szeged.hu (Z.K.); kakos@chem.u-szeged.hu (Á.K.); 4 MTA-SZTE Reaction Kinetics and Surface Chemistry Research Group, H-6720 Szeged, Hungary; 5 Department of Inorganic and Analytical Chemistry, University of Szeged, Dóm tér 7, H-6720 Szeged, Hungary; 6 Department of Physical Chemistry and Materials Science, University of Szeged, H-6720 Szeged, Hungary

**Keywords:** cysteine oxo acids, cysteinate oxidation to cystinate, cysteinate anion-pillared CaAl-layered double hydroxide (LDH), confined space—molecular reactor

## Abstract

L-Cysteinate-intercalated CaAl-layered double hydroxide (LDH) was prepared by the co-precipitation method producing highly crystalline hydrocalumite phase with a well-pillared interlayer gallery. The obtained materials were characterized by X-ray diffractometry, IR as well as Raman spectroscopies. By performing interlamellar oxidation reactions with peracetic acid as oxidant, oxidation of cysteinate to cystinate in aqueous and cysteinate sulfenic acid in acetonic suspensions occurred. The oxidations could be performed under mild conditions, at room temperature, under neutral pH and in air. It has been shown that the transformation pathways are due to the presence of the layered structure, that is, the confined space of the LDH behaved as molecular reactor.

## 1. Introduction

Sulfur-containing organic compounds—especially cysteine and its derivatives—as bioactive ingredients or functional bioconjugates have been widely applied in chemo-enzymatic syntheses as well as composing biomedicines for many years [1,2,3]. Numerous previous studies demonstrated considerable improvement in the biocatalytic activity of living and artificial enzymes containing thiol- and/or disulfide-containing redox moieties [4,5,6]. Additionally, biomimetic compounds modelled after natural enzymes including cystine side chains proved to be efficient biosensors and serodiagnostic test molecules as well as catalysts in fermentation applications [7,8,9,10,11]. The oxo acid forms of cysteine also play significant roles as medicines or nucleophilic partners in enantioselective reactions from, for instance, nitrile synthesis to the treatment of plantar hyperkeratosis or acne [12,13,14,15].

Unfortunately, in spite of these useful applications, aiming the production of these derivatives through the oxidation of cysteine remains synthetic challenge because of the numerous feasible reaction pathways and possible side products [16,17]. It has been shown that transformations of cysteine and cysteine-related molecules towards various products can be achieved via the development of catalytic reaction systems and/or exploiting the interlayer gallery of layered materials are needed [18,19,20,21,22].

The challenge of widening the palette of ways for the lab-scale oxidative transformations of cysteine motivated our work. The idea was to anchor the anionic form of cysteine among the layers of CaAl-LDH (hydrocalumite) and probe various oxidizing agents. This combination has never been tried before. In the followings, it is shown that by tuning the reaction conditions and altering the solvent, influencing the transformation pathways in a beneficial way became possible.

## 2. Materials and Methods

### 2.1. Materials

l-cysteine, Ca(NO_3_)_2_ × 4H_2_O, Al(NO_3_)_3_ × 9H_2_O, NaOH, peracetic acid, bromine, hydrogen peroxide, methanol and acetone were purchased from Sigma Aldrich (Budapest, Hungary) and were used without further purification.

### 2.2. Synthesis of Nitrate-Containing and L-Cysteinate-Intercalated CaAl-LDH

A modified co-precipitation method was used for the synthesis and the intercalation process. Firstly, the organic compounds (most often 6.0 × 10^−3^ mol, but 1.2 × 10^−2^ or 1.8 × 10^−2^ mol were also used) were dissolved in 65 cm^3^ of methanol, then 100 cm^3^ of aqueous solution containing Ca(NO_3_)_2_ × 4H_2_O (0.3 M) and Al(NO_3_)_3_ × 9H_2_O (0.15 M) was added, and the pH of 13.1 was set with NaOH solution (3 M). The mixture was stirred at 65 °C for 3 days. Then the suspension was filtered, washed with water and methanol several times and dried at 85 °C overnight. Nitrate-containing LDH (layered double hydroxide) was produced in the same way without adding organic phase to the slurry. All operations were performed under nitrogen atmosphere to minimize carbonate intercalation from the airborne CO_2_.

### 2.3. Interlayer Oxidation of L-Cysteinate

**Scheme 1:**
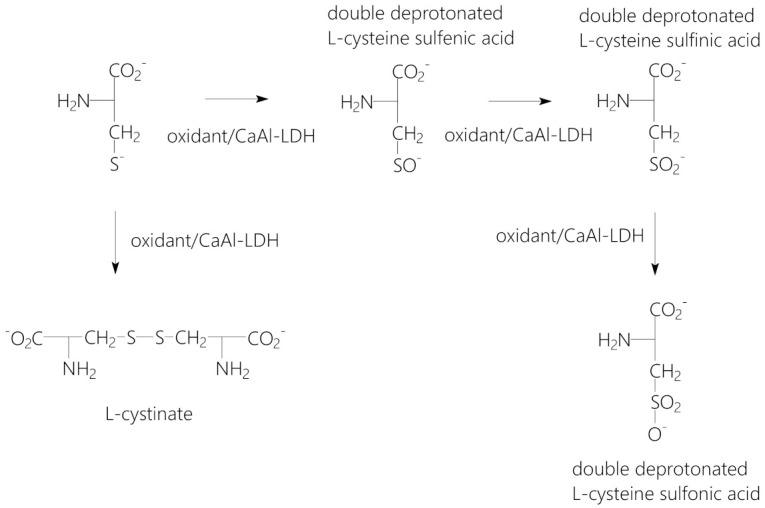
Possible reaction pathways of L-cysteinate oxidation incorporated in CaAl-LDH.

During the oxidation process, the oxidizing agent (peracetic acid, bromine or hydrogen peroxide) was applied in various concentrations (from 0.4 M to 0.7 M) adding them dropwise to the suspensions containing the intercalated LDH suspended in water, methanol or acetone (100 cm^3^). The suspensions were stirred for 60 min, then they were filtered and dried at 353 K. The amount of the intercalated LDH (0.1 g) and the temperature (298 K) were the same in all cases. The possible reaction pathways are depicted in Scheme 1.

### 2.4. Methods of Structural Characterization

Powder X-ray patterns were recorded by a Rigaku XRD-6000 diffractometer (Rigaku, Tokyo, Japan) using CuKα radiation (λ = 0.15418 nm) at 40 kV and 30 mA with 2°/min scan speed.

The instrument for recording the IR spectra was a Bruker Vertex 70 spectrophotometer (Bruker, Billerica, MA, USA) equipped with a single reflection diamond ATR accessory (Bruker, Billerica, MA, USA). Spectra were recorded in the 4000–400 cm^−1^ wavenumber range, but only the most informative wavenumber ranges will be displayed and discussed in the paper. 256 scans were collected for each spectrum, and the resolution was 4 cm^−1^.

Raman spectra were recorded with a Raman Senterra II (Bruker) microscope (Bruker, Billerica, MA, USA) at an excitation wavelength of 765 nm applying 12.5 mW laser power and averaging 20 spectra with an exposition time of 20 s.

UV/Vis spectroscopy was used for the quantitative analysis of l-cysteine at the wavelength of 231 nm. The members of the calibration series as well as the unknown samples were measured on a Shimadzu UV-1650 spectrophotometer (Shimadzu, Kyoto, Japan).

## 3. Results and Discussions

**Figure 1:**
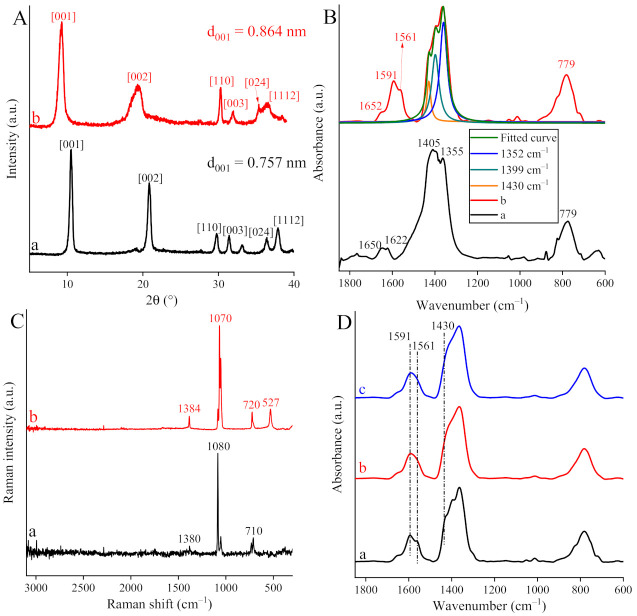
X-ray diffraction (XRD) patterns (**A**), IR (**B**) and Raman (**C**) spectra of CaAl–NO_3_^–^–LDH (a) and CaAl–cysteinate–LDH (b). IR spectra (**D**) of CaAl–cysteinate–LDH (0.1 g) using 6.0 × 10^−^^3^ mol (a), 1.2 × 10^−^^2^ mol (b) or 1.8 × 10^−^^2^ mol (c) intercalant.

After preparing CaAl-LDH in the well-known co-precipitation synthesis route, through combining previously published intercalation methods [21,22,23], cysteinate anions were immobilized in the interlayer gallery of hydrocalumite (CaAl-LDH). The success of this operation was verified by X-ray diffraction (XRD) measurements (Figure 1A) and the incorporated amount was 8 × 10^−5^ mol. Both the as-prepared and intercalated structures exhibited diffraction patterns corresponding to lamellar monoclinic structure in P2_1_ space group being analogous to that of a nitrate-containing CaAl-LDH (JCPDS database: PDF #89-6723). Furthermore, the (00l) and the other diffraction lines of intercalated hydrocalumite shifted to smaller angles compared to those of the as-prepared one indicating the expansion of basal spacing from 0.757 nm to 0.864 nm associated with altering the anions in the interlayer space. The extent of the expansion corresponds to single-layer insertion of the cysteinate anions, which is in good agreement with previous reports [21,22,23].

The as-prepared LDH and the intercalated composite were studied by IR as well as Raman spectroscopies (Figure 1B,C).

The band due to the ν3 (asymmetric stretching) mode of nitrate anions at 1355 cm^−1^ could be seen in IR spectrum of the as-prepared LDH along with ν3 vibration band of surface-adsorbed carbonate (1405 cm^−1^) and β(OH) vibration band (1650 cm^−1^) as well as the characteristic vibration band identified as the stretching vibration mode of Al–O bond (779 cm^−1^) [24]. The IR spectrum of the composite material exhibited bands characteristic of an LDH with intercalated carboxylate anions [25,26]. Accordingly, fingerprint-like vibration modes of the carboxylate group appeared, the asymmetric vibrations bands at ~1600 and 1569 cm^−1^ and the symmetric one at ~1433 cm^−1^. In accordance with literature data, doubling of ν_as_(COO^−^) vibration suggested that cysteinate anions were linked to the layers as syn-anti carboxylate bridges with bidentate carboxylate coordination [27]. During intercalation, partial anion exchange only occurred indicated by the “survival” of peaks attributed to carbonate and nitrate ions. In view of the absence of the significant changes in the IR spectra, the smallest amount of cysteinate (6.0 × 10^–3^ mol to 0.1 g of LDH) was adequate for achieving the maximum extent of ion exchange. (Figure 1D).

On one hand, Raman spectra also indicated partial intercalation, as the characteristic Raman bands of nitrate/carbonate ions (~1380, 1070–1080, 710–720 cm^−1^), were not eliminated. On the other hand, the appearance of a S–C stretching band (527 cm^−1^) evidenced the presence cysteinate anions [28]. Additionally, the lack of the intense stretching vibration of sulfhydryl group (~2500 cm^−1^) directly demonstrated that thiol groups were deprotonated during the intercalation facilitating the charge neutrality of the composite materials.

**Figure 2:**
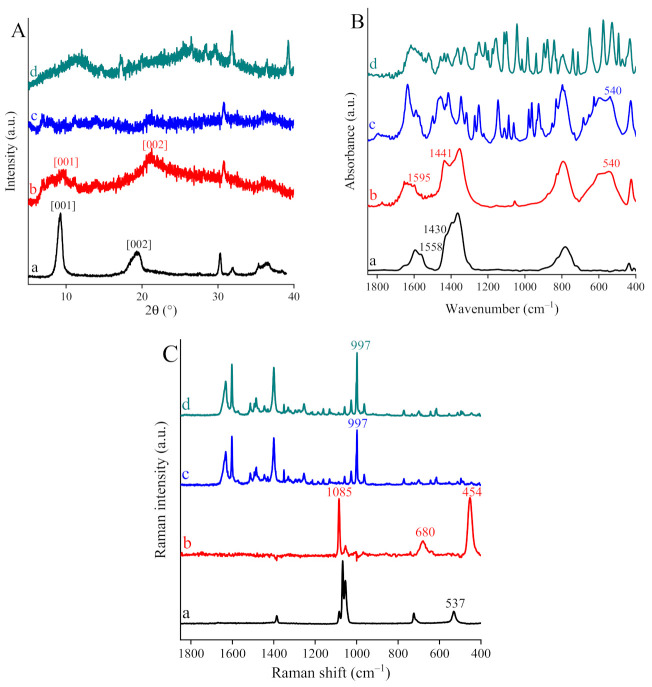
XRD patterns (**A**), IR (**B**) and Raman (**C**) spectra of CaAl–cysteinate–LDH (a) and CaAl–cysteinate–LDH (0.1 g) oxidized by 0.4 M of peracetic acid (b), bromine (c) or H_2_O_2_ (d) suspended in water (100 cm^3^) at 298 K. The reaction time was 60 min.

In order to efficiently select a suitable reactant for the interlayer oxidation of the cysteinate ions for more detailed studies, scouting experiments were performed using peracetic acid, bromine or H_2_O_2_ at 298 K using water as solvent. The XRD patterns (Figure 2A) attested that both bromine and hydrogen peroxide damaged the layered structure, which resulted in uncontrollable product distribution. Many products were formed, which was indicated by the IR and Raman spectra (Figure 2B,C) having so many bands that selecting the main products was impossible. The only exception was the case of peracetic acid, which oxidized cysteinate to cystinate, and, although the crystallinity was decreased the LDH reflections could be identified. The formation of cystinate was best highlighted by the intense sharp peak at 454 cm^−1^ and the less intense at 680 cm^−1^, assigned to the stretching vibration modes of C–S and S–S bonds, respectively [29]. Additionally, new bands at 1441 and 1595 cm^−1^ could be detected in the IR spectrum revealing changes in the local environment of the carboxylate groups [30,31].

**Figure 3:**
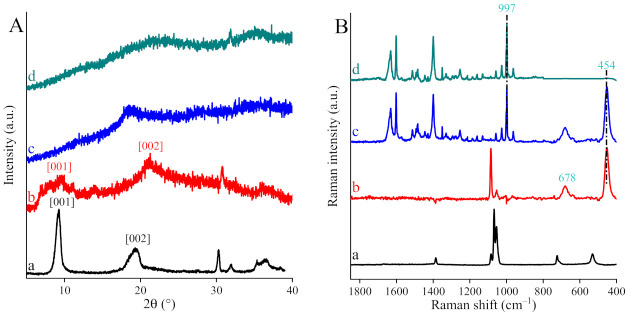
XRD patterns (**A**) and Raman (**B**) spectra of CaAl–cysteinate–LDH (a) and CaAl–cysteinate–LDH oxidized by peracetic acid; reaction conditions: the oxidizing agent in 0.4 M (b), 0.55 M (c) or 0.70 M (d) concentration was applied to 0.1 g of composite suspended in 100 cm^3^ of water at 298 K. The reaction time was 60 min.

Upon increasing the amount of peracetic acid, the layered structure disappeared indicating the complete collapse of hydrocalumite structure (Figure 3A, traces c and d) leading to uncontrollable product distribution once again (Figure 3B, traces c and d). Clearly, the presence of the layered structure was crucial for the oxidation to occur [32].

**Figure 4:**
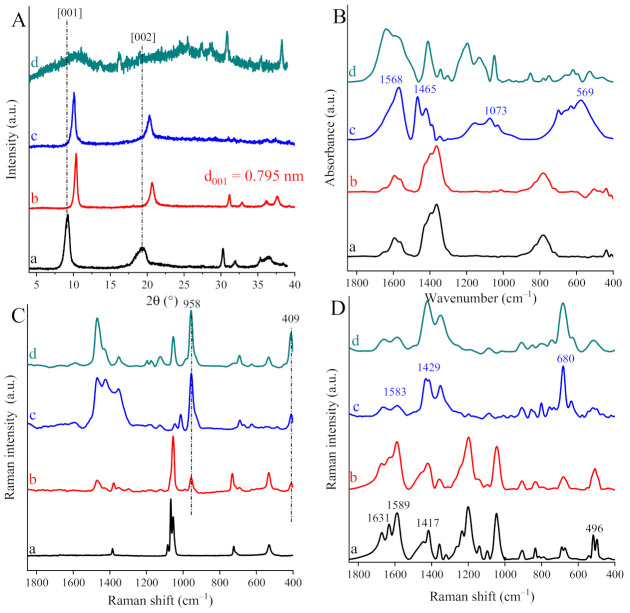
XRD patterns (**A**), IR (**B**) and Raman spectra (**C**) of CaAl–cysteinate–LDH (a) and CaAl–cysteinate–LDH (0.1 g) oxidized by peracetic acid using 0.4 M (b), 0.55 M (c) and 0.70 M (d) peracetic acid in methanol (100 cm^3^). Raman spectra (**D**) of cysteine (1 g) oxidized by peracetic acid (0.4 M) in methanol (100 cm^3^) with (a) or without (b) LDH (0.1 g) as additive as well as in acetone (100 cm^3^) with (c) or without (d) LDH (0.1 g) as additive at 298 K. The reaction time was 60 min.

On replacing water with organic solvents (acetone or methanol), notable changes in the product distribution were observed. In methanol, the oxidation resulted in significantly lower extent in the degradation of LDH structure than it was experienced in aqueous medium (Figure 4A). On detecting relatively high shift in the characteristic (00*l*) reflections, significant decrease in the interlayer space occurred, quite the opposite of the expectations. This finding called our attention to another possible transformation pathway. The IR spectrum of the composite exhibited some strong absorptions over the whole measurement range with bands differing from those of cystinate (Figure 4B). Intense bands in the range of 1600–1400 cm^−1^ were assigned to antisymmetric and symmetric absorption bands of cysteinate derivatives, while less intense bands centered at 1073 cm^−1^ could be assigned to the in-plane carboxylic acid COH bending mode of cysteine sulfonic acid, consistent with previous assignments [33,34]. Furthermore, the band position and intensity of the stretching vibration bands of C–S band were changed substantially indicating the formation of one or more cysteine oxo acid forms. On the basis of the observations described above, the formation of oxo acid anion mixture is suggested, the main product being double deprotonated cysteine sulfonic acid anions along with double deprotonated cysteine sulfenic acid anions or, as it was intuitively rather expected, double deprotonated cysteine sulfinic acid anions. Much to our surprise, the Raman spectra of the composite suggested the formation of double deprotonated cysteine sulfenic acid anions without any doubt (Figure 4C). The absorption band at 958 cm^−1^, which increased in intensity in parallel with the increase in the concentration of the oxidant, was associated with the stretching vibration mode of deprotonated S–O(H) functional group of cysteine sulfenic acid [35]. Furthermore, there was no evidence that S–O_2_^–^ functional group of cysteine sulfinate was produced, this group should have had two intense broad bands in the range of 1250–1000 cm^−1^ [35]. By performing the oxidation in non-aqueous solvents without the LDH or dosing it as additive to the solution, it was not possible to produce any of the desired products selectively (Figure 4D). In all cases, both cysteinate and cystinate as well as oxo acid forms were produced. Accordingly, the presence of the layered material providing confined space as molecular reactor with fixed cysteinate ion in it was a contribution of utmost significance in facilitating the oxidation in a beneficial way.

**Figure 5:**
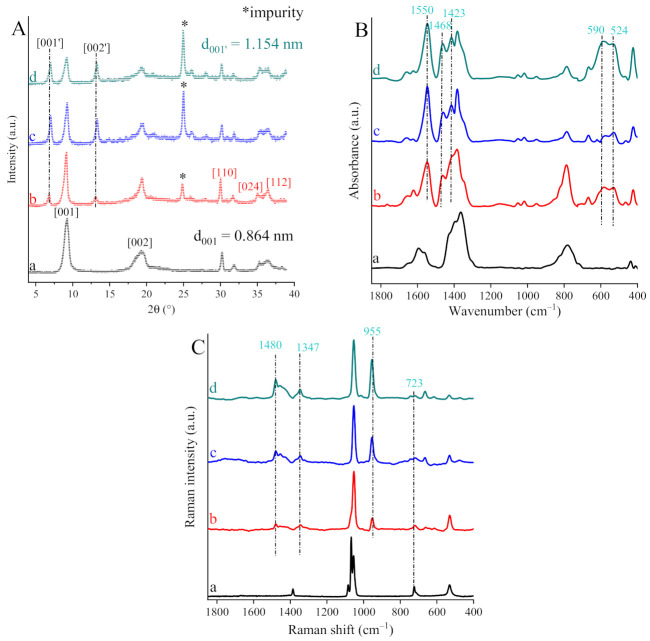
XRD patterns (**A**), IR (**B**) and Raman spectra (**C**) of CaAl–cysteinate–LDH (a) and CaAl–cysteinate–LDH (0.1 g) oxidized by peracetic acid upon using 0.4 M (b), 0.55 M (c) and 0.65 M (d) peracetic acid in acetone (100 cm^3^) at 298 K.

Finally, by repeating the screening in acetone, a completely different transformation pathway was observed. The ordered structure of LDH was not lost during oxidation, irrespective to the concentration of peracetic acid verified by the XRD patterns (Figure 5A). Moreover, a new LDH phase was developed without eliminating the original one, that is, staging occurred. The interlayer gallery for the new phase was enlarged (1.154 nm) compared to the cysteinate-containing counterpart (0.864 nm). Additionally, the ratio of new LDH phase to the original one grew in line with the increasing amount of added peracetic acid. (It is fair to mention though that a relatively strong and sharp reflection related to unidentified impurity arose at about 25° of 2θ values, the intensity of which increased with increasing concentration of peracetic acid. Since the other reflections are assigned to LDH structures, it is reasonable to assume that certain amount of the product formed was released from the interlayer space, and it might be adsorbed on the outer surface of the LDH resulting in the unidentified reflection.).

A series of absorption bands in IR spectra appeared at 1550, 1468, 1423 and 590 as well as 524 cm^−1^, they could be clearly attributed to the characteristic vibration modes of cysteine sulfenic acid [33]. Distinct strong absorbances in the high-energy region (1620–1420 cm^−1^) agreed well with the antisymmetric and symmetric vibration modes of carboxylate group overlapped with vibrations of sulfenyl group, while less intense bands at about 550 cm^−1^ could be related to the deformation mode vibrations of C–S bond. No evidence for the formation of cysteine sulfinate were found. Including strong peaks at 955, 1347 and 1480 cm^−1^ identified as stretching vibration mode of deprotonated S–O(H) and carboxylate functional groups [35], Raman spectra of the composites provided with clear, fingerprint-like evidence for the exclusive production of double deprotonated cysteine sulfenic acid anions fixed among the layers of CaAl-LDH.

In our view, the changes observed in the oxidation products on altering the solvent is due to the changes of hydration/dehydration state of the interlayer gallery. Water is an integral part of an LDH, attached to the wall of the layers and to each other by secondary, probably mainly hydrogen bonds. On changing the solvent from water to methanol or acetone, at least partial replacement of the interlayer water molecules took place, thus, the immediate environment for the reaction was modified significantly, while all the other parameters (the LDH, reaction temperature, reaction time, the oxidant) remained unaltered.

## 4. Conclusions

Applying CaAl-LDH as a molecular reactor, intercalated cysteinate anions could be oxidized with peracetic acid to various products depending on the solvent used. The formation of cystinate was experienced by using water as solvent, while double deprotonated cysteine sulfenic acid anions was observed in acetone solvent. The crucial contribution of the layered structure to the observed ways of transformations was evidenced.

Also, it was reasonable to assume that the hydration/dehydration state of the interlayer space modified by the solvents induced the significant shift in the oxidation pathways.

## Data Availability

Data are available with the authors.
